# Supplementary material for: Splice-Junction-Based Mapping of Alternative Isoforms in the Human Proteome
Source: Cell Rep. Author manuscript; Available in PMC 2020 Jan 15. (PMC6961840; doi:10.1016/j.celrep.2019.11.026)
Supplement: 2 [file NIHMS1546469-supplement-2.zip › DF1/PXD006675/RightCoronaryArtery/RightCoronaryArtery_6_TNXB_MGPLSVVIVTVAR.pdf]

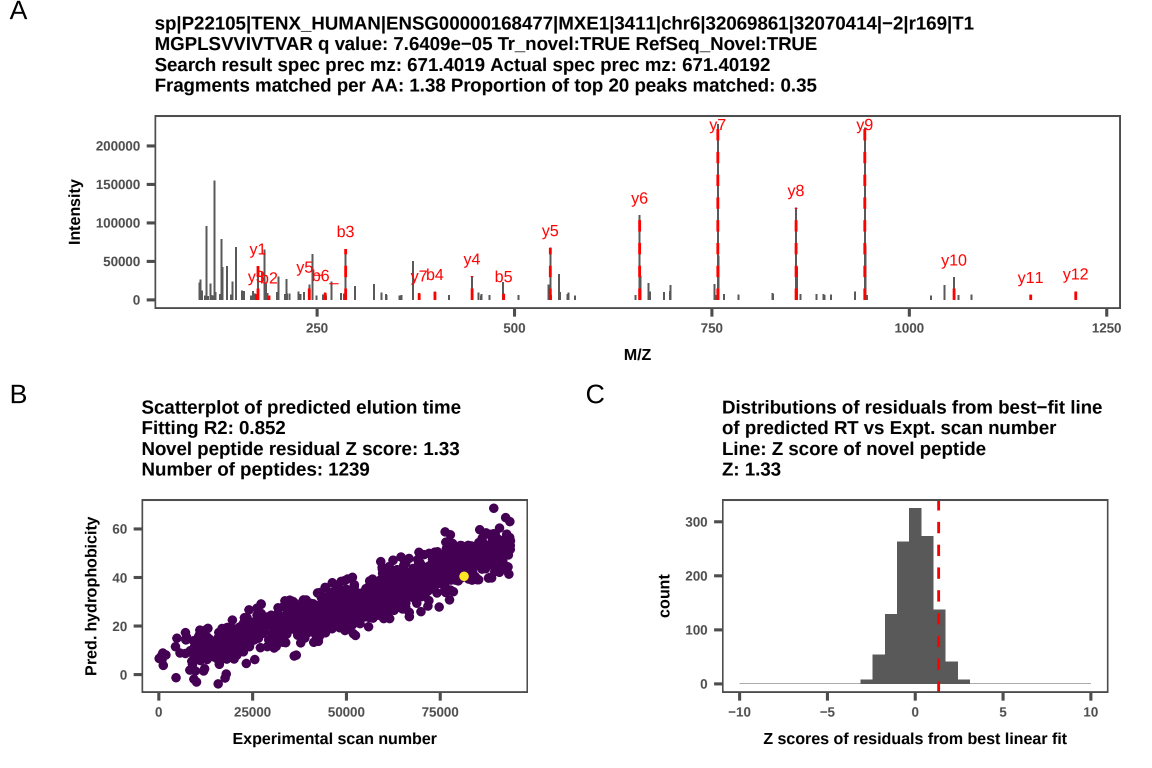
A
sp|P22105|TENX_HUMAN|ENSG00000168477|MXE1|3411|chr6|32069861|32070414|−2|r169|T1
MGPLSVVIVTVAR q value: 7.6409e−05 Tr_novel:TRUE RefSeq_Novel:TRUE
Search result spec prec mz: 671.4019 Actual spec prec mz: 671.40192
Fragments matched per AA: 1.38 Proportion of top 20 peaks matched: 0.35
Intensity
200000
150000
100000
50000
0
y7
y9
y6
y8
b3
y5
y1
y4
y10
y5
b6
y7
b4
b5
y11
y12
b2
250
500
750
1000
1250
M/Z
B
Scatterplot of predicted elution time
Fitting R2: 0.852
Novel peptide residual Z score: 1.33
Number of peptides: 1239
Pred. hydrophobicity
60
40
20
0
25000
50000
75000
Experimental scan number
C
Distributions of residuals from best−fit line
of predicted RT vs Expt. scan number
Line: Z score of novel peptide
Z: 1.33
count
300
200
100
0
−10
−5
0
5
10
Z scores of residuals from best linear fit
